# A Suggestion for Secretaries

**Published:** 1889-02-09

**Authors:** 


### EDITOR'S LETTER-BOX.

[Our Correspondents are reminded that prolixity is a great bar to publication, and that brevity of style and conciseness of statement greatly facilitate early insertion.]

### A SUGGESTION FOR SECRETARIES.

"Charity-boxes," as you observe in your last issue, are very common objects in hospitals, but very little money ever finds its way into them. Dozens of cases receive treatment in the casualty ward—often for weeks running—and when the house surgeon pronounces them well out they walk without even a "thank you, sir;" and certainly without dropping a penny-piece into the box, though it stands on the table in full view, with the request in large white letters, "Please give 1d." Neither are the "casualty patients" the only people to find fault with. In the board-room stands a box, also in full view, and also the same request, "Please give," but I can assert for the last six months not one sixpence has entered the slit. We should be many a pound richer if only the many visitors who call in and out of hours, and who take up so much of a matron's valuable time, oftentimes asking most foolish questions, would put but sixpence into the box, we should be thankful. Hospitals must be supported, but how? For £1 1s. subscribers so often think a patient can be kept *ad lib.* If churches and chapels would give without requests, if subscribers would only pay early in the year, much money in printing and sending out notices would be saved; no collector would be needed, consequently no per cent. on new sums. The public are always ready to be down on hospital expenses, forgetting they themselves cause many of them.—A. E. S.

